# Technical Evaluation: Identification of Pathogenic Mutations in *PKD1* and *PKD2* in Patients with Autosomal Dominant Polycystic Kidney Disease by Next-Generation Sequencing and Use of a Comprehensive New Classification System

**DOI:** 10.1371/journal.pone.0166288

**Published:** 2016-11-11

**Authors:** Moritoshi Kinoshita, Eiji Higashihara, Haruna Kawano, Ryo Higashiyama, Daisuke Koga, Takafumi Fukui, Nobuhisa Gondo, Takehiko Oka, Kozo Kawahara, Krisztina Rigo, Tim Hague, Kiyonori Katsuragi, Kimiyoshi Sudo, Masahiko Takeshi, Shigeo Horie, Kikuo Nutahara

**Affiliations:** 1 Diagnostic Division, Otsuka Pharmaceutical Co., Ltd., Tokushima, Japan; 2 Department of ADPKD Research, School of Medicine, Kyorin University, Tokyo, Japan; 3 Department of Urology, School of Medicine, Kyorin University, Tokyo, Japan; 4 Department of Urology, Graduate School of Medicine, Juntendo University, Tokyo, Japan; 5 FALCO biosystems Ltd., Kyoto, Japan; 6 World Fusion Co., Ltd., Tokyo, Japan; 7 Omixon Ltd., Budapest, Hungary; 8 Samon-cho Clinic, Medical Corporation Shinanokai, Tokyo, Japan; Central South University, CHINA

## Abstract

Genetic testing of *PKD1* and *PKD2* is expected to play an increasingly important role in determining allelic influences in autosomal dominant polycystic kidney disease (ADPKD) in the near future. However, to date, genetic testing is not commonly employed because it is expensive, complicated because of genetic heterogeneity, and does not easily identify pathogenic variants. In this study, we developed a genetic testing system based on next-generation sequencing (NGS), long-range polymerase chain reaction, and a new software package. The new software package integrated seven databases and provided access to five cloud-based computing systems. The database integrated 241 polymorphic nonpathogenic variants detected in 140 healthy Japanese volunteers aged >35 years, who were confirmed by ultrasonography as having no cysts in either kidney. Using this system, we identified 60 novel and 30 known pathogenic mutations in 101 Japanese patients with ADPKD, with an overall detection rate of 89.1% (90/101) [95% confidence interval (CI), 83.0%–95.2%]. The sensitivity of the system increased to 93.1% (94/101) (95% CI, 88.1%–98.0%) when combined with multiplex ligation-dependent probe amplification analysis, making it sufficient for use in a clinical setting. In 82 (87.2%) of the patients, pathogenic mutations were detected in *PKD1* (95% CI, 79.0%–92.5%), whereas in 12 (12.8%) patients pathogenic mutations were detected in *PKD2* (95% CI, 7.5%–21.0%); this is consistent with previously reported findings. In addition, we were able to reconfirm our pathogenic mutation identification results using Sanger sequencing. In conclusion, we developed a high-sensitivity NGS-based system and successfully employed it to identify pathogenic mutations in *PKD1* and *PKD2* in Japanese patients with ADPKD.

## Introduction

Polycystic kidney disease (PKD) is one of the most common inherited disorders affecting the renal tubules. It comprises autosomal dominant PKD (ADPKD) and autosomal recessive PKD [1–3]. Around 85% of patients with ADPKD have mutations in *PKD1* (16p13.3) and around 15% have mutations in *PKD2* (4q22.1) [4–6]. Mutations in *PKD1* lead to end-stage renal disease (ESRD) by, on average, age 53.3 years, which is earlier than the average age of ESRD in patients with *PKD2* mutations (72.7 years) [7].

Diagnosis of ADPKD is usually based on family history, ultrasonography, computed tomography (CT), or magnetic resonance imaging (MRI) [8]. Genetic testing, however, can facilitate the diagnosis in patients whose renal phenotypes are unclear and in patients for whom there is lack information regarding family history; it may also help identify donors for renal transplantation [9]. However, modern genetic testing methods are currently not part of the standard of care. This is partly because of the difficulties in testing *PKD1* and *PKD2* by conventional direct sequencing methods such as Sanger sequencing because there is a high degree of allelic heterogeneity in both *PKD1* and *PKD2* and their combined coding regions are quite long, amounting to 61 exons to analyze (46 in *PKD1* and 15 in *PKD2*), and there are six *PKD1* pseudogenes that share a high degree of homology with most of *PKD1* [10, 11].

To improve the sequencing shortfalls, long-range polymerase chain reaction (LR-PCR) that can amplify all the exonic regions with several sets of primers was developed. As previously reported, the LR-PCR method required five different PCR conditions to amplify the 46 exons of *PKD1*. This was simpler than direct sequencing, but the procedure was still complex, and *PKD2* was not assessed [12]. Thus, we developed unique primers, and combinations thereof, to amplify all the *PKD1* and *PKD2* exons simultaneously under similar PCR conditions, thereby simplifying the testing procedure for these genes.

Recently, several next-generation sequencing (NGS) platforms have been approved for *in vitro* diagnostic devices, indicating that genetic testing using NGS may have an important role to play in clinical testing in the near future. The overall mutation detection rate of *PKD* gene analysis using older NGS methods was reported to be lower than that using the Sanger method [13]; however, in recent articles, high sensitivity has been reported [14–17]. In our new method for detecting genetic mutations in *PKD1* and *PKD2*, the latest benchtop NGS machine was used for the analysis of large genes; NGS was considered suitable for *PKD* genetic testing because of its high-throughput capability.

For the detection and identification of pathogenic mutations in *PKD1* and *PKD2* in each patient, a novel software package was developed to perform the analysis. This software package integrated seven databases, including the polymorphic variants detected in 140 healthy Japanese volunteers. An efficient and comprehensive genetic testing system based on LR-PCR, an NGS platform, and the software package was evaluated by testing 101 Japanese families with ADPKD to identify the pathogenic genetic mutations in all of the patients.

## Materials and Methods

### Participants and Materials

A total of 101 unrelated patients with ADPKD, all age 19 years or older, were recruited at Kyorin University Hospital (N = 82) and Juntendo University Hospital (N = 19) in Japan from 2014 to 2015. ADPKD was diagnosed by imaging, in accordance with a previous report [18]. In addition, 140 healthy Japanese volunteers were recruited at the Medical Corporation Shinanokai, Samoncho Clinic, Tokyo, Japan. Volunteers were age 35 or older and were confirmed, by ultrasonography, as having no renal cysts. The experimental protocol was reviewed and approved by the following local ethics committees: Independent Ethics Committee of Kyorin University (Approval study ID: Kyorin-PKD-1), Independent Ethics Committee of Juntendo University Hospital (Approval number: 13–151) and Independent Research Ethics Committee of Otsuka Pharmaceutical Co., Ltd. (Approval number: 131007 and 131217), and written informed consent was obtained from all participants; the study was submitted and registered in the ClinicalTrials.gov registry (Identifier: NCT02322385). Genomic DNA from lymphocytes was extracted from 6 mL peripheral blood using the QIAamp DNA Mini Kit (QIAGEN, Venlo, Netherlands) at FALCO Biosystems Ltd in Kyoto, Japan, and was stored at 4°C until use.

### LR-PCR

We designed LR-PCR primers to amplify 18 long DNA fragments, including the exonic regions of *PKD1* and *PKD2*. These fragments were amplified from participants’ purified genomic DNA using the primers shown in S1 Table. Multiple LR-PCR products were amplified in each of the following combinations: A (LR-PCR 2 and 12), B (4 and 17), C (6 and 15), D (1, 5, and 9), E (3, 8, and 18), F (7, 13, and 16), and G (10, 11, and 14). The LR-PCR reactions were performed simultaneously on the same PCR plate using the following touchdown PCR regimen [19]: (i) 94°C for 2 min; (ii) one cycle at 98°C for 10 s and 74°C for 5 min; (iii) one cycle at 98°C for 10 s and 72°C for 5 min; (iv) one cycle at 98°C for 10 s and 70°C for 5 min; (v) 30 cycles at 98°C for 10 s and 68°C for 5 min; and (vi) 68°C for 7 min. The LR-PCR products were purified using the Agencourt AMPure XP kit (Beckman Coulter, Inc, Brea, CA, USA) and quantified as previously described [20].

With the Ion PGM instrument (Thermo Fisher Scientific Inc, Waltham, MA, USA), only weak coverage could be achieved for the *PKD1* exon 1 region because it contains a GC-rich sequence. To circumvent this problem, corrective PCR was performed in which *PKD1* exon 1 was re-amplified from the combination D LR-PCR product noted above, using the primers shown in S2 Table. The PCR products were purified according to the procedure described above.

### Sequencing

#### NGS by Ion PGM

The LR-PCR and corrective PCR products were used to prepare libraries with an Ion Xpress Plus Fragment Library Kit (Thermo Fisher Scientific Inc). In each assay batch, the LR-PCR–derived libraries and corrective PCR-derived libraries from six patients were mixed at a ratio of 7:3 to yield barcoded libraries, each of which had a total nucleotide concentration of 26 pmol/L in the mixture. After treating the samples using an Ion PGM Template OT2 Kit (Thermo Fisher Scientific Inc) according to the manufacturer’s protocol [21], emulsion PCR was performed using the Ion OneTouch 2 System (Thermo Fisher Scientific Inc), followed by enrichment of the beads using Ion OneTouch ES (Thermo Fisher Scientific Inc). The enriched emulsion-PCR products were prepared for sequencing using an Ion PGM Sequencing 200 Kit v2 (Thermo Fisher Scientific Inc) and then loaded onto an Ion 318 v2 chip (Thermo Fisher Scientific Inc) and sequenced with an Ion PGM.

#### NGS by MiSeq

In preparation for sequencing on a MiSeq sequencer (Illumina, Inc, San Diego, CA, USA), the LR-PCR products from seven patients were mixed and subjected to fragmentation processing to obtain fragments measuring approximately 300 bp; adaptor/tag sequences were added using a Nextera XT Kit (Illumina, Inc) according to the manufacturer’s instructions [22]. Next, the fragments were processed to amplify clusters in a paired-end flow-cell and sequenced on a MiSeq System according to the paired-end method with 150 bp read length, using MiSeq Reagent Kit v2 (Illumina, Inc).

#### Sanger Sequencing

In preparation for Sanger sequencing, the LR-PCR products were purified using an Agencourt AMPure XP kit (Beckman Coulter, Inc). They were then subjected to cycle sequencing with BigDye Terminator v1.1 Cycle Sequencing Kit (Thermo Fisher Scientific Inc) and purified using BigDye Xterminator Purification Kit (Thermo Fisher Scientific Inc) [23]. The sequencing products were subjected to electrophoresis and sequenced on a 3130xl Genetic Analyzer (Thermo Fisher Scientific Inc). SeqScape Software v2.6 (Thermo Fisher Scientific Inc) was used to detect mutations based on comparisons with reference sequences (*PKD1*: NG_008617, *PKD2*: NG_008604) [24].

### Mutation Detection System

**Fig 1:**
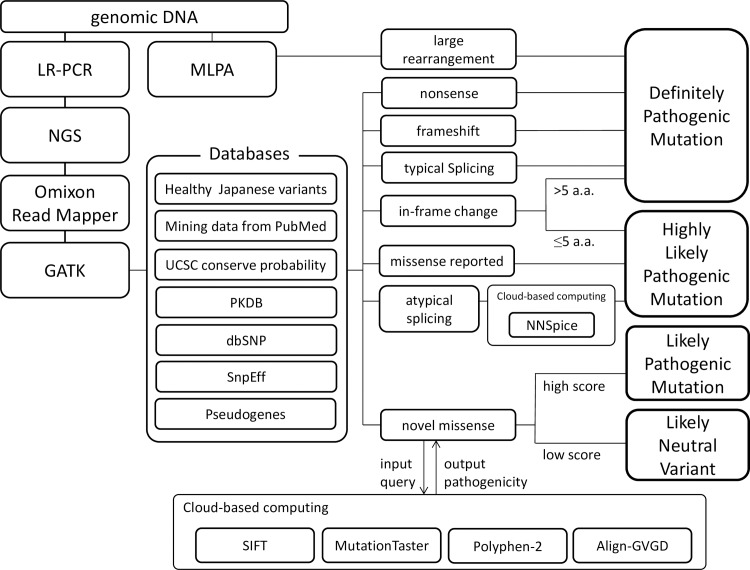
Schematic representation of the pipeline for identification of pathogenic mutations. Pathogenic mutations were identified on the basis of seven annotated databases: PKDB, dbSNP, SnpEff, UCSC (for conservation probability), PubMed (article searches), Pseudogene.org, and a database of polymorphic variants in 140 healthy Japanese individuals. Novel missense mutations and potential splicing mutations were evaluated for pathogenicity using public cloud-based computing (SIFT, PolyPhen-2, Align-GVGD, MutationTaster, and NNSplice). PKDB; PKD mutation database, NCBI; National Center for Biotechnology Information, dbSNP; Single Nucleotide Polymorphism database, UCSC; University of California, Santa Cruz, SIFT; Sorting Intolerant from Tolerant, GVGD; Grantham Variation Grantham Deviation.

Unique and specific analysis software was customized with Omixon Target (Omixon Ltd, Budapest, Hungary) to identify pathogenic genetic mutations in either *PKD1* or *PKD2*, exclusively (Fig 1). Omixon Target is a software package for analyzing targeted sequencing data obtained from NGS platforms [25]. The software was set up to use hg19 [26] as the primary human reference sequence, and the target region was set to detect variants in all exons of *PKD1* and *PKD2* by extending the margins 30 bp beyond the exon–intron boundaries. This software package performed mapping and alignment of read sequences onto reference sequence hg19 using the Omixon-Read-Mapper software (Omixon Ltd) with imported FASTQ files from the sequencing instrument. After mapping, genomic variants were called using a toolkit for genome analysis from GATK (Broad Institute, Cambridge, MA, USA).

The called mutations were annotated using our customized database of variants in 140 healthy Japanese volunteers and databases reconstructed from the Single Nucleotide Polymorphism database (dbSNP) of the National Center for Biotechnology Information (NCBI) [27], the PKD mutation database (PKDB) [28], the predicted effects of SnpEff [29], and mutation variants reported in PubMed. The”position and base pattern match”rule was applied; that is, an annotation was applied if the position, reference, and actual mutation coincided for the variant and the annotation in each database. Other databases of conservation probability and pseudogene annotation were applied if the patterns matched at a position. The conservation probability data was sourced from the University of California, Santa Cruz (UCSC) Vertebrate Conservation Score [30] for the relevant regions of *PKD1* and *PKD2*. This software package had a graphical user interface and ran on Windows.

### Mutation Classification

With reference to the classification protocol described by Rossetti et al. [11] and Audrézet et al. [31], the mutations detected in this study were categorized into four classes: definitely pathogenic, highly likely to be pathogenic, likely pathogenic, and likely neutral. Nonsense mutations, frameshift mutations, large rearrangements detected by multiplex ligation-dependent probe amplification (MLPA) analysis, typical splicing mutations previously confirmed as truncating mutations in PubMed literature, and in-frame changes of more than five amino acids were all classified as definitely pathogenic mutations. Missense mutations that had been reported previously in patients with ADPKD, atypical splicing mutations predicted to be splicing defects on the basis of public cloud-based computing program NNSplice [32] detection of nucleotide substitution mutations in the vicinity of exon–intron junctions, and in-frame changes of fewer than six amino acids were classified as highly likely pathogenic mutations. Novel missense mutations predicted as pathogenic by public cloud-based analyses including Sorting Intolerant from Tolerant (SIFT) [33], PolyPhen-2 [34], Align Grantham Variation Grantham Deviation (A-GVGD) [35] and MutationTaster [36] were classified as likely pathogenic mutations, according to the scoring method of Genkyst in a previous study [31]. Missense mutations with no predicted pathogenicity and silent mutations were classified as likely neutral variants (Fig 1).

### MLPA Analysis

Samples that were found to be mutation-negative by NGS were subjected to MLPA analysis [37]. We screened for large rearrangements involving deletions and duplications in *PKD1* and *PKD2* using multiple PCR reactions for each exon. The SALSA MLPA *PKD1* (P351), *PKD2* (P352), and *TSC2* (P046) kits were purchased from MRC-Holland, Inc (Amsterdam, Netherlands). After denaturation of sample DNA, a mixture of 105 MLPA probes was added to the sample. For each exon sequence of the sample DNA, two adjacent MLPA probes were hybridized and ligated into a single probe with DNA ligase. All ligated probes on each exon were amplified simultaneously using a common PCR primer pair. One PCR primer was labeled with a fluorescent tag and the resulting PCR amplicons were visualized using capillary electrophoretic separation. Deletion of one or more exon sequence in the sample DNA was identified by a decrease in peak height, which reflects amplification.

## Results

### Specific Amplification of *PKD1* and *PKD2* by LR-PCR

The LR-PCR products were sufficiently amplified as single bands visualized by 1% agarose gel electrophoresis (S1 Fig). All of the LR-PCR reactions were performed simultaneously using the same conditions and in the same plate.

### Construction of a Database of Polymorphisms in Healthy Volunteers

**Fig 2:**
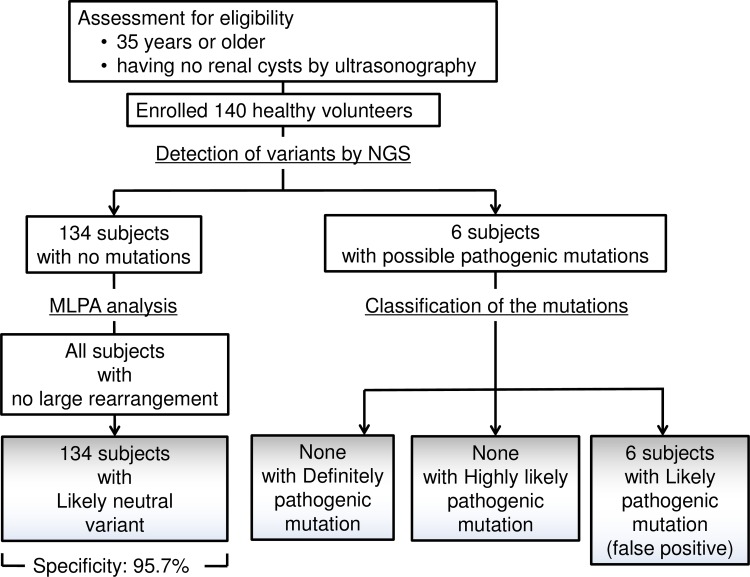
Schematic diagram of workflow in healthy volunteers. 140 healthy Japanese volunteers were recruited and they were age 35 or older and were confirmed as having no renal cysts by ultrasonography. Four nonsynonymous variants predicted to be likely pathogenic mutations in six subjects and other 134 subjects were predicted in likely neutral variants by the scoring protocol using cloud-based computing [31]. The specificity of the system was estimated to be 95.7%.

All of the 140 healthy Japanese volunteers were imaged by ultrasonography to confirm absence of cysts. Based on the analysis of all healthy subjects using the variant identification/classification criteria, 241 nucleotide variants were detected in *PKD1* and *PKD2* (S3 Table). Four nonsynonymous variants (p.R3183Q was detected in three subjects and p.A3879T, p.R1587C, and p.G1395R were detected in one subject) were predicted to be likely pathogenic mutations and the other 237 were predicted to be likely neutral variants, according to the scoring protocol using cloud-based computing. From these results, the specificity of our system was estimated to be 95.7% (134/140) (Fig 2). The 237 nonpathogenic variants were then assembled into a database comprising polymorphic variants of healthy Japanese volunteers. This database was loaded onto the *PKD1/2* variant analysis system with other reconstructed databases, including PKDB, dbSNP, Pseudogene.org, UCSC conservation probability, and mutations previously collected from PubMed articles.

### Identification of Pathogenic Mutations in Patients with ADPKD

S2 Fig shows representative mapping patterns of some of the pathogenic mutations detected in this study, such as missense mutations and typical splicing mutations and frameshift mutations caused by nucleotide(s) deletion and insertion. MLPA analysis was performed on DNA from 11 patients with no pathogenic mutations in *PKD1* or *PKD2* detected by LR-PCR analysis; four cases of large rearrangements were detected.

**Fig 3:**
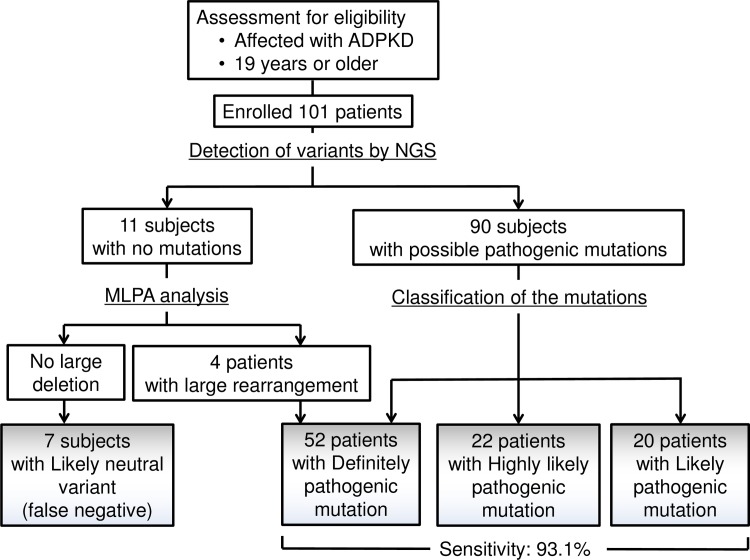
Schematic diagram of workflow in the patients with ADPKD. 52 definitely pathogenic mutations, 22 highly likely pathogenic mutations and 20 likely pathogenic mutations were identified in 101 Japanese patients with ADPKD. The sensitivity of the system was estimated to be 93.1% in combined with multiplex ligation-dependent probe amplification analysis (MLPA).

We detected 663 mutations, including polymorphic variants, in 101 Japanese patients with ADPKD; among these mutations, we identified those predicted to be pathogenic (Fig 3).

Details of the pathogenic mutations according to the three classification types are shown in S4–S6 Tables. Definitely pathogenic mutations, highly likely pathogenic mutations, and likely pathogenic mutations were identified in 52 (55.3%), 14 (14.9%), and 28 (29.7%) patients, respectively (Table 1). There were 82 pathogenic mutations detected in *PKD1* [87.2%; 95% confidence interval (CI), 80.5%–94.0%] and 12 detected in *PKD2* (12.8%; 95% CI, 6.0%–19.5%). Thus, the ratio of pathogenic mutations in *PKD1* relative to *PKD2* agreed with previous studies [38, 39]. In addition, the positive predictive value (PPV) and negative predictive value (NPV) were 94.0% (94/100) and 95.0% (134/141), respectively.

**Table 1:** Summary of the pathogenic mutations in Japanese patients with ADPKD.

Pathogenic mutation		*PKD1*		*PKD2*		Total		
Definitely Pathogenic mutation						52 (55.3)		
	Nonsense mutation	10		3				13 (14.1)
	Frameshift mutation	29		4				33 (35.9)
	Typical splicing mutation	2		0				2 (2.2)
	Large rearrangements	4		0				4 (4.3)
Highly likely pathogenic mutation						14 (14.9)		
	Missense mutation reported	8		3			11 (12.0)	
	In-frame change	3		0			3 (3.3)	
	Atypical splicing mutation	6		2			8 (8.7)	
Likely pathogenic mutation						28 (29.7)		
	Novel missense mutation	20		0			20 (21.7)	
Total (%)		82	(87.2)	12	(12.8)		94 (100)	

At Kyorin University hospital, 78 of 82 (95.1%; 95% CI, 90.5%–99.8%) patients with ADPKD were identified with pathogenic mutations, and at Juntendo University hospital, 16 of 19 (84.2%; 95% CI, 67.8%–100%) patients with ADPKD were identified with pathogenic mutations. No significant differences were found in the mutation detection rates between the two hospitals. The four large rearrangements detected by MLPA analysis were not characterized by nucleotide sequence, because MLPA analysis can detect the presence of each exon. Both the novel and known pathogenic mutations identified in this study are summarized in Table 2.

**Table 2:** Summary of novel and known pathogenic mutations identified in this study.

(1) Definitely pathogenic mutation			
Mutation	Gene	Novel pathogenic mutation	Known pathogenic mutation
Nonsense mutation	*PKD1*	p.Q149X, p.W1893X, p.Y2388X, p.W3936X	p.R1436X, p.E2458X, p.W3603X, p.Q3962X, p.Q4216X, p.Q4238X
	*PKD2*	p.Y311X, p.Y836X	p.R654X
Frameshift mutation	*PKD1*	p.G233fs25X (2)*, p.G282fs5X, p.P414fs50X, p.L1459fs74X, p.N1474fs48X, p.V1542fs34X, p.S1876fs68X, p.R1951fs5X, p.Q2305fs10X, p.R2310fs26X, p.Q2784fs37X, p.P2790fs83X, p.P2809fs65X, p.V2897fs39X, p.L3033fs39X, p.A3171fs142X, p.E3252fs6X, p.A3987fs53X, p.G4205fs155X,	p.S1457fs64X, p.R1672fs98X (4)*, p.R1990fs58X, p.R2643fs10X, p.L2650fs9X, p.L3656fs28X
	*PKD2*	p.R139fs93X, p.F436fs3X, p.V489fs36X, p.S701fs14X	None
Typical splicing	*PKD1*	None	p.D97_I120del (c.288-2A>G) p.Q2305fs10X (c.6916-9G>A)
	*PKD2*	None	None
Large rearrangements	*PKD1*	exon29-30del, exon3del, exon40del, exon3del	None
	*PKD2*	None	None
(2) Highly likely pathogenic mutation			
Mutation	Gene	Novel pathogenic mutation	Known pathogenic mutation
Missense mutation reported	*PKD1*	None	p.L727P (2)*, p.W967R, p.A3136V, p.S3599L, p.R3753W (2)*, p.A4002G
	*PKD2*	None	p.R322Q (3)*
In-frame change ≤ 3 amino acid	*PKD1*	p.L3287del (2)*	p.R2765_S2766ins4
	*PKD2*	None	None
Atypical splicing	*PKD1*	p.I120fs32X, p.R400fs29X, p.L1054fs71X, p.P1098fs4X, p.K3607_V3608ins24, p.S3904fs123X	None
	*PKD2*	p.V516fs87X, p.R786fs5X	None
(3) Likely pathogenic mutation			
Mutation	Gene	Novel pathogenic mutation	Known pathogenic mutation
Novel missense mutation	*PKD1*	p.R380C (2)*, p.E574G, p.W1414C, p.G2034E, p.G2034R, p.C2178Y, p.Y2228C, p.D2660N, p.R2681C, p.C3043R, p.G3116V, p.G3127S, p.R3269Q, p.R3753G, p.G3818D, p.Q3838K, p.E4025D, p.E4148D (2)*	None
	*PKD2*	None	None

*Identical mutations are indicated by numbers in parentheses. These numbers denote the number of patients who have the same mutation.

### Comparison with Sanger Sequencing and Other NGS Methods

Sanger sequencing is regarded as the gold standard for sequencing. To validate our system, Sanger sequencing was performed on DNA from 20 patients randomly selected from those we identified as having definite pathogenic mutations. Their mutations were confirmed by the Sanger method. In addition, our system could be applied using a MiSeq sequencer (another NGS sequencing method). The pathogenic mutations identified in these 20 patients by Ion PGM sequencing and MiSeq sequencing were identical.

## Discussion

Launched in 2011, NGS has become a popular medical tool because of its convenient benchtop method, cost-effectiveness, and suitability for targeted sequencing [40]. Benchtop NGS instruments are now used in routine genetic testing in reference laboratories. They can be used even for large genes such as *PKD1* or *PKD2* because of their high-throughput analytic capacity. For these reasons, a convenient ADPKD testing system based on NGS was developed for clinical use.

Six pseudogenes that share a high degree of homology with *PKD1* could hinder the performance of genetic testing of *PKD1*. The LR-PCR primers in this study were designed to avoid misamplification of these pseudogenes. This system allowed all of the LR-PCR reactions to run simultaneously under the same conditions on the same instrument. This improvement simplifies the LR-PCR procedure.

Despite extensive efforts to differentiate pathogenic mutations from unclassified variants [11, 31], it has been difficult to establish whether a genetic change is a pathogenic mutation or a polymorphism. In this study, we improved the system for distinguishing pathogenic mutations from nonpathogenic variants by following three steps: first, we combined the most up-to-date databases of *PKD* pathogenic mutations worldwide; second, we incorporated the data of 237 normal polymorphic variants from 140 healthy Japanese volunteers into the combined databases; third, we confirmed the results obtained from the combined databases using well-established bioinformatic tools to predict pathogenic relevance by cloud-based computing.

Our NGS system in combination with the software and LR-PCR achieved high sensitivity with MLPA analysis—an overall detection rate of 93.1% among 101 Japanese patients with ADPKD. Rossetti et al. also analyzed DNA samples from patients with ADPKD using NGS, in 2012, and reported an overall detection rate of 63% [13]. This low detection rate may be related to the insufficient performance of NGS sequencers at that time and the short read length of 75 bp used in their protocol. In our study, we used a read length of 200 bp with the latest method and sequencer. It is advantageous to use long read lengths for detecting variations such as insertion/deletion mutations. Furthermore, using the same samples, the pathogenic mutation results obtained by Sanger sequencing were found to be consistent with those obtained using our system. When our system was tested using a MiSeq sequencer applied to the same LR-PCR products, the pathogenic mutations identified were the same as those obtained using the Ion PGM. Thus, our system can work effectively with different NGS platforms. Previously it has been reported that the performance of the Ion PGM is lower than that of the MiSeq [41]. However, according to our results, even a mutation that comprised a 76 bp deletion (G4205indel; 12613_12690del77insA) could be detected using the Ion PGM; thus, its performance can be considered on par with standard sequencing methods.

Recent improvements in technology have led to NGS studies with favorable sensitivities of >90% [15–17]. However, the sensitivity of 99.2% reported by Tan et al [15] was the matching rate between the variants obtainedfrom 25 patients detected by their NGS and those detected previously by Sanger method. Further, they calculated the specificity in a manner similar to that of the reference alleles. Eisenberger et al [16] also evaluated the specificity that was the matching rate of the polymorphic variants from 55 patients between NGS and Sanger method. In contrast, our study evaluated the clinical sensitivity of the pathogenic mutations using 101 patients and we originally evaluated the clinical specificity using 140 volunteers who were confirmed as being healthy.

In a comprehensive analysis of Japanese patients with ADPKD, Kurashige et al. detected genetic mutations in *PKD2* at a rate of 23.6% based on the Sanger sequencing method [42]. They reported that the frequency of mutations in *PKD2* was significantly higher than in European patients with ADPKD. However, we identified genetic mutations in *PKD2* at a rate of 12.6% in Japanese patients with ADPKD recruited at two independent hospitals. The different population ratio might be attributable to population-selection bias. Renal function survival in *PKD1* mutation carriers is 15–20 years shorter than in *PKD2* mutation carriers [7, 43]; therefore, patient selection at clinics where there are many patients with ESRD might lead to a higher proportion of *PKD1* mutation carriers and conversely a lower proportion of *PKD2* mutation carriers.

The identical mutations found in two or more unrelated patients were as follows: p.G233fs25X, p.R380C, p.L727P, p.R1672fs98X, p.L3287del, p.R3753W, and p.E4148D in the *PKD1* gene and p.R322Q in the *PKD2* gene. The genealogy of all the ADPKD patients was investigated and no connections between their pedigrees were identified, confirming them to be genetically unrelated. Thus, it is likely that these identical mutations arose as independent recurrent mutations, though we cannot rule out the possibility that they were derived from founder mutations originating from common remote ancestors. The p.R1672fs98X (c.5014-5015delAG) mutation in *PKD1* was the most frequent recurrent mutation—observed in four unrelated patients. This mutation was reported to be a typical recurrent mutation arising through the mechanism of non-homologous end joining [31].

The detection rate of large rearrangements is reported to be about 4% in patients with ADPKD [44]. Previously, it was recommended that MLPA analysis be performed first, in *PKD* gene mutation analysis, because the Sanger sequencing method was both complicated and expensive. However, our results indicate that this recommendation should be updated. Because the NGS system developed in this study is cost-effective and has high-throughput performance, the pair–end mapping feature in the genotyping protocol makes it possible to detect such large deletions located within the LR-PCR amplicon [16], MLPA analysis would be recommended only for those patients who emerge as being mutation-negative following sequencing with this NGS system.

Among the four nonsynonymous variants found in our sample of 140 healthy individuals (and initially classified as likely pathogenic mutations), variant p.R3183Q was found in three people. Thus, it is likely that this variant is a normal polymorphic variant in the population; it has been filed in dbSNP as rs79648977 and in PKDB as “indeterminate.” On the other hand, variant p.G1395R has been filed as “likely pathogenic mutation” in PKDB, and the other two variants have no annotations. These variants were detected in healthy individuals (one person each) who had no renal cysts evident by ultrasonography. Because these volunteers were 35 years of age or older, the possibility of late-onset ADPKD emerging cannot be formally ruled out at this time. Ongoing follow-up of these individuals could provide further important information that will be helpful for future genetic analysis and understanding of ADPKD.

In conclusion, we developed an improved and efficient genetic testing system for ADPKD based on NGS, incorporating the easy-to-use LR-PCR approach to avoid amplification of pseudogenes and a newly developed software system to effectively identify pathogenic mutations. For Japanese patients with ADPKD, the sensitivity of this new system was 93.1%, specificity was 95.7%, and PPV and NPV were 94.0% and 95.0%, respectively. We anticipate that our system will facilitate genetic testing of ADPKD.

## Supporting Information

S1 Fig Ethidium bromide staining of LR-PCR products electrophoresed on a 1% agarose gel. The LR-PCR products were electrophoresed on a 1% agarose gel. Each set of LR-PCR amplicons in a given lane had been amplified in the same well. All of the multiple LR-PCR reaction combinations were performed simultaneously using the same PCR machine. Lane M1: λDNA/HindIII marker; Lane A: combination A of LR-PCR product 2 (3,610 bp) and 12 (2,418bp); Lane B: combination B of LR-PCR products 4 (5,356 bp) and 17 (2,949 bp); Lane C: combination C of LR-PCR products 6 (3,555 bp) and 15 (4,516 bp); Lane D: combination D of LR-PCR products 1 (2,547 bp), 5 (2,579 bp), and 9 (3,559 bp); Lane E: combination E of LR-PCR products 3 (3,703 bp), 8 (3,079 bp), and 18 (1,516 bp); Lane F: combination F of LR-PCR products 7 (3,727 bp), 13 (4,172 bp), and 16 (1,534 bp); Lane G: combination G of LR-PCR products 10 (1,561 bp), 11 (1,240 bp), and 14 (1,206 bp); Lane M2: 1 kb DNA ladder (TAKARA Bio Inc, Kyoto, Japan).
(TIF)

S2 Fig Mapping and alignment analysis of several representative pathogenic genetic mutations detected in patients with ADPKD. The upper gray bars show the depth of coverage and the lower red and blue lines show the mapped read fragments. (a) Missense mutation 9379G > A (G3127S) in *PKD1*, (b) splicing mutation IVS2-2A > G (D97_I120del24) in *PKD2*, (c) deletion 9755_9756delAG (E3252fs6X) in *PKD1*, and (d) insertion 8689insC (V2897fs39X) in *PKD1*.
(TIF)

S1 Table Sequences of oligonucleotide primers for LR-PCR and their locations in the *PKD1* or *PKD2* gene. (XLS)

S2 Table Sequences and positions of oligonucleotide primers for corrective PCR re-amplification of *PKD1* exon 1. (XLS)

S3 Table Nucleotide variants detected in 140 Japanese healthy volunteers. (XLS)

S4 Table Details of definitely pathogenic mutations of *PKD1* and *PKD2* in Japanese patients with ADPKD. (XLS)

S5 Table Details of highly likely pathogenic mutations of *PKD1* and *PKD2* in Japanese patients with ADPKD. (XLS)

S6 Table Details of likely pathogenic mutations of *PKD1* and *PKD2* in Japanese patients with ADPKD. (XLS)

## References

[1] Gabow PA. Autosomal dominant polycystic kidney disease. New Engl J Med. 1993;329: 332–342. 10.1056/NEJM199307293290508 8321262

[2] Grantham JJ. Polycystic kidney disease: neoplasia in disguise. Am J Kidney Dis. 1990;15: 110–116. 10.1016/S0272-6386(12)80507-5 2405652

[3] Higashihara E, Nutahara K, Kojima M, Tamakoshi A, Yoshiyuki O, Sakai H, et al Prevalence and renal prognosis of diagnosed autosomal dominant polycystic kidney disease in Japan. Nephron 1998;80: 421–427. 10.1159/000045214 9832641

[4] The European Polycystic Kidney Disease Consortium. The polycystic kidney disease 1 gene encodes a 4kb transcript and lies within a duplicated region on chromosome 16. Cell 1994;77: 881–894. 10.1016/0092-8674(94)90137-6 8004675

[5] Mochizuki T, Wu G, Hayashi T, Xenophontos SL, Veldhuisen B, Saris JJ, et al PKD2, a gene for polycystic kidney disease that encodes an integral membrane protein. Science 1996;272: 1339–1342. 10.1126/science.272.5266.1339 8650545

[6] Dicks E, Ravani P, Langman D, Davidson WS, Pei Y, Parfrey PS. Incident renal events and risk factors in autosomal dominant polycystic kidney disease: a population and family-based cohort followed for 22 years. Clin J Am Soc Nephrol. 2006;1: 710–717. 10.2215/CJN.01581105 17699277

[7] Torra R, Badenas C, Donell A, Nicolau C, Volpini V, Revert L, et al Linkage, clinical features, and prognosis of autosomal dominant polycystic kidney disease type 1 and 2. JASN 1996;7: 2142–2151. 8915974 10.1681/ASN.V7102142

[8] Thomsen HS, Levine E, Meilstrup JW, Van Slyke MA, Edgar KA, Barth JC, et al Renal cystic diseases. Eur Radiol. 1997;7: 1267–1275. 10.1007/s003300050288 9377514

[9] Huang E, Picotal MS, McCune T, Melancon JK, Montgomery RA, Ugarte R, et al DNA testing for live kidney donors at risk for autosomal dominant polycystic kidney disease. Transplantation 2009;87: 133–137. 10.1097/TP.0b013e318191e729 19136903 PMC2841023

[10] Bogdanova N, Markoff A, Gerke V, McCluskeyc M, Horsta J, Dworniczaka B. Homologues to the first gene for autosomal dominant polycystic kidney disease are pseudogenes. Genomics 2001;74: 333–341. 10.1006/geno.2001.6568 11414761

[11] Rossetti S, Consugar MB, Chapman AB, Torres VE, Guay-Woodford LM, Grantham JJ, et al Comprehensive molecular diagnostics in autosomal dominant poly cystic kidney disease. JASN 2007;18: 2143–2160. 10.1681/ASN.2006121387 17582161

[12] Tan YC, Michaeel A, Blumenfeld J, Donahue S, Parker T, Levine D, et al A novel long-range PCR sequencing method for genetic analysis of entire PKD1 gene. J Mol Diag. 2012;14: 305–313. 10.1016/j.jmoldx.2012.02.007 22608885 PMC3391417

[13] Rossetti S, Hopp K, Sikkink RA, Sundsbak JL, Lee YK, Kubly V, et al Identification of gene mutations in autosomal dominant polycystic kidney disease through targeted resequencing. JASN 2012;23: 915–933. 10.1681/ASN.2011101032 22383692 PMC3338301

[14] Yang T, Meng Y, Wei X, Shenc J, Zhangb M, Qia C, et al Identification of novel mutations of PKD1 gene I Chinese patients with autosomal dominant polycystic kidney disease by targeted next-generation sequencing. Clin Chim Acta. 2014;443: 12–19. 10.1016/j.cca.2014.02.011 24582653

[15] Eisenberger T, Decker C, Hiersche M, Hamann RC, Decker E, Neuber S, et al An efficient and comprehensive strategy for genetic diagnostics of polycystic kidney disease. PLoS ONE 2015;10: e0116680 10.1371/journal.pone.0116680 25646624 PMC4315576

[16] Tan YC, Michaeel A, Genyan L, Elemento, Blumenfeld J, Donahue S, et al Molecular Diagnosis of autosomal dominant polycystic kidney disease using next-generation sequencing. J Mol Diag. 2014;16: 216–228. 10.1016/j.jmoldx.2013.10.005 24374109 PMC3937536

[17] Trujillano D, Bullich G, Ossowski, Ballarín J, Torra R, Estivill X, et al Diagnosis of autosomal dominant polycystic kidney disease using efficient PKD1 and PKD2 targeted next-generation sequencing. Mol Genet Genomic Med. 2014;2: 412–421. 10.1002/mgg3.82 25333066 PMC4190876

[18] Pei Y, Obaji J, Dupuis A, Paterson AD, Magistroni R, Dicks E, et al Unified criteria for ultrasonographic diagnosis of ADPKD. JASN 2009;20: 205–212. 10.1681/ASN.2008050507 18945943 PMC2615723

[19] Don RH, Cox PT, Wainwright BJ, Baker K, Mattick JS. ‘Touchdown’ PCR to circumvent spurious priming during gene amplification. Nucleic Acids Res. 1991;19: 4008 10.1093/nar/19.14.4008 1861999 PMC328507

[20] Rodrigue S, Materna AC, Timberlake SC, Blackburn MC, Malmstrom RR, Alm EJ, et al Unlocking short read sequencing for metagenomics. PLoS ONE 2010;5: e11840 10.1371/journal.pone.0011840 20676378 PMC2911387

[21] Vogel U, Szczepanowski R, Claus H, Jünemannb S, Priorb K, Harmsen D. Ion torrent personal genome machine sequencing for genomic typing of Neisseria meningitidis for rapid determination of multiple layers of typing information. J Clin Biol. 2012;50: 1889–1894. 10.1128/JCM.00038-12 22461678 PMC3372157

[22] De novo bacterial sequencing on the MiSeq system. Illumina Application Note. 2011. Available: http://www.illumina.com/content/dam/illumina-marketing/documents/products/appnotes/appnote_miseq_denovo.pdf#search='De+novo+bacterial+sequencing+on+the+MiSeq+system.'.

[23] Regalado ES, Guo DC, Villamizar C, Avidan N, Gilchrist D, McGillivray B, et al Exome sequencing identifies SMAD3 mutations as a cause of familial thoracic aorticaneurysm and dissection with intracranial and other arterial aneurysms. Circ Res. 2011;109: 680–686. 10.1161/CIRCRESAHA.111.248161 21778426 PMC4115811

[24] Casari G, Sander C, Valencia A. A method to predict functional residues in proteins. Nature Structural Biol. 1995;2: 171–178. 10.1038/nsb0295-171 7749921

[25] Oliver GR. Considerations for clinical read alignment and mutational profiling using next-generation sequencing. F1000Res. 2012 7 16;1: 2 10.12688/f1000research.1-2.v2 24627757 PMC3945748

[26] http://hgdownload-test.cse.ucsc.edu/goldenPath/hg19/phastCons46way/vertebrate/.

[27] Sherry ST, Ward MH, Kholodov M, Baker J, Phan L, Smigielski EM, et al dbSNP: the NCBI database of genetic variation. Nucleic Acids Res. 2001;29: 308–311. 10.1093/nar/29.1.308 11125122 PMC29783

[28] Autosomal Dominant Polycystic Kidney Disease Mutation Database: http://pkdb.mayo.edu/index.html.

[29] Siepel A, Bejerano G, Pedersen JS, Hinrichs AS, Hou M, Rosenbloom K, et al Evolutionarily conserved elements in vertebrate, insect, worm, and yeast genomes. Genome Res. 2005; 15: 1034–1050. 10.1101/gr.3715005 16024819 PMC1182216

[30] GATK Guide article. Adding genome annotations using SnpEff VariantAnnotator. Available: http://www.broadinstitute.org/gatk/guide/ updated 10 June, 2013.

[31] Audrézet MP, Cornec-Le Gall E, Chen JM, Redon S, Quéré I, Creff J, et al Autosomal dominant polycystic kidney disease: comprehensive mutation analysis of PKD1 and PKD2 in 700 unrelated patients. Hum Mutat. 2012;33: 1239–1250. 10.1002/humu.22103 22508176

[32] Reese MG, Eeckman FH, Kulp D, Haussler D. Improved Splice Site Detection in Genie. J Comp Biol. 2009;4: 311–323. 10.1089/cmb.1997.4.311 9278062

[33] Ng PC, Henikoff S. SIFT: predicting amino acid changes that affect protein function. Nucleic Acids Res. 2003;31: 3812–3814. 10.1093/nar/gkg509 12824425 PMC168916

[34] Polyphen2: prediction of functional effects of human nonsynonymous SNPs. Available: http://genetics.bwh.harvard.edu/pph2/

[35] Tavtigian SV, Greenblatt MS, Lesueur F, Byrnes GB. In silico analysis of missense substitutions using sequence-alignment based methods. Hum Mutat. 2008;29: 1327–1336. 10.1002/humu.20892 18951440 PMC3431198

[36] Schwarz JM, Rödelsperger C, Schuelke M, Seelow D. MutationTaster evaluates disease-causing potential of sequence alterations. Nat Methods. 2010;7: 575–576. 10.1038/nmeth0810-575 20676075

[37] Schouten JP, McElgunn CJ, Waaijer R, Zwijnenburg D, Diepvens F, Pals G. Molecular profiling of invasive breast cancer by multiplex ligation-dependent probe amplification-based copy number analysis of tumor suppressor and oncogenes. Nucleic Acids Res. 2002;30: e57 10.1093/nar/gnf056 12060695 PMC117299

[38] Higashihara E, Torres VE, Chapman AB, Grantham JJ, Bae K, Watnick TJ, et al Tolvaptan in autosomal dominant polycystic kidney disease: Three years’ experience. Clin J Am Soc Nephrol. 2011;6: 2499–2507. 10.2215/CJN.03530411 21903984 PMC3359559

[39] Torres VE, Chapman AB, Devuyst O, Gansevoort RT, Grantham JJ, Higashihara E, et al Tolvaptan in patients with autosomal dominant polycystic kidney disease. N Engl J Med. 2012;367: 2407–2418. 10.1056/NEJMoa1205511 23121377 PMC3760207

[40] Mardis ER. A decade’s perspective on DNA sequencing technology. Nature 2011;470: 198–203. 10.1038/nature09796 21307932

[41] Quail MA, Smith M, Coupland P, Otto TD, Harris SR, Connor TR, et al A tale of three next-generation sequencing platforms: comparison of Ion Torrent, Pacific Biosciences and Illumina MiSeq sequencers. BMC Genomics. 2012;13: 341 10.1186/1471-2164-13-341 22827831 PMC3431227

[42] Kurashige M, Hanaoka K, Imamura M, Udagawa T, Kawaguchi Y, Hasegawa T, et al A comprehensive search for mutations in the PKD1 and PKD2 in Japanese subjects with autosomal dominant polycystic kidney disease. Clin Genet 2015;87: 266–272. 10.1111/cge.12372 24611717

[43] Hateboer N, v Dijk MA, Bogdanova N, Coto E, Saggar-Malik AK, San Millan JL, et al Comparison of phenotypes of polycystic kidney disease types 1 and 2. European PKD1-PKD2 Study Group. Lancet 1999;353: 103–107. 10.1016/S0140-6736(98)03495-3 10023895

[44] Consugar MB, Wong WC, Lundquist PA, Rossetti S, Kubly VJ, Walker DL, et al Characterization of large rearrangements in autosomal dominant polycystic kidney disease and the PKD1/TSC2 contiguous gene syndrome. Kidney Int. 2008;74: 1468–1479. 10.1038/ki.2008.485 18818683 PMC2756756

