# Supplementary figures and images for: Technical Evaluation: Identification of Pathogenic Mutations in PKD1 and PKD2 in Patients with Autosomal Dominant Polycystic Kidney Disease by Next-Generation Sequencing and Use of a Comprehensive New Classification System
Source: PLoS One. 2016 Nov 11;11(11):e0166288. doi: 10.1371/journal.pone.0166288 (PMC5105999; doi:10.1371/journal.pone.0166288)

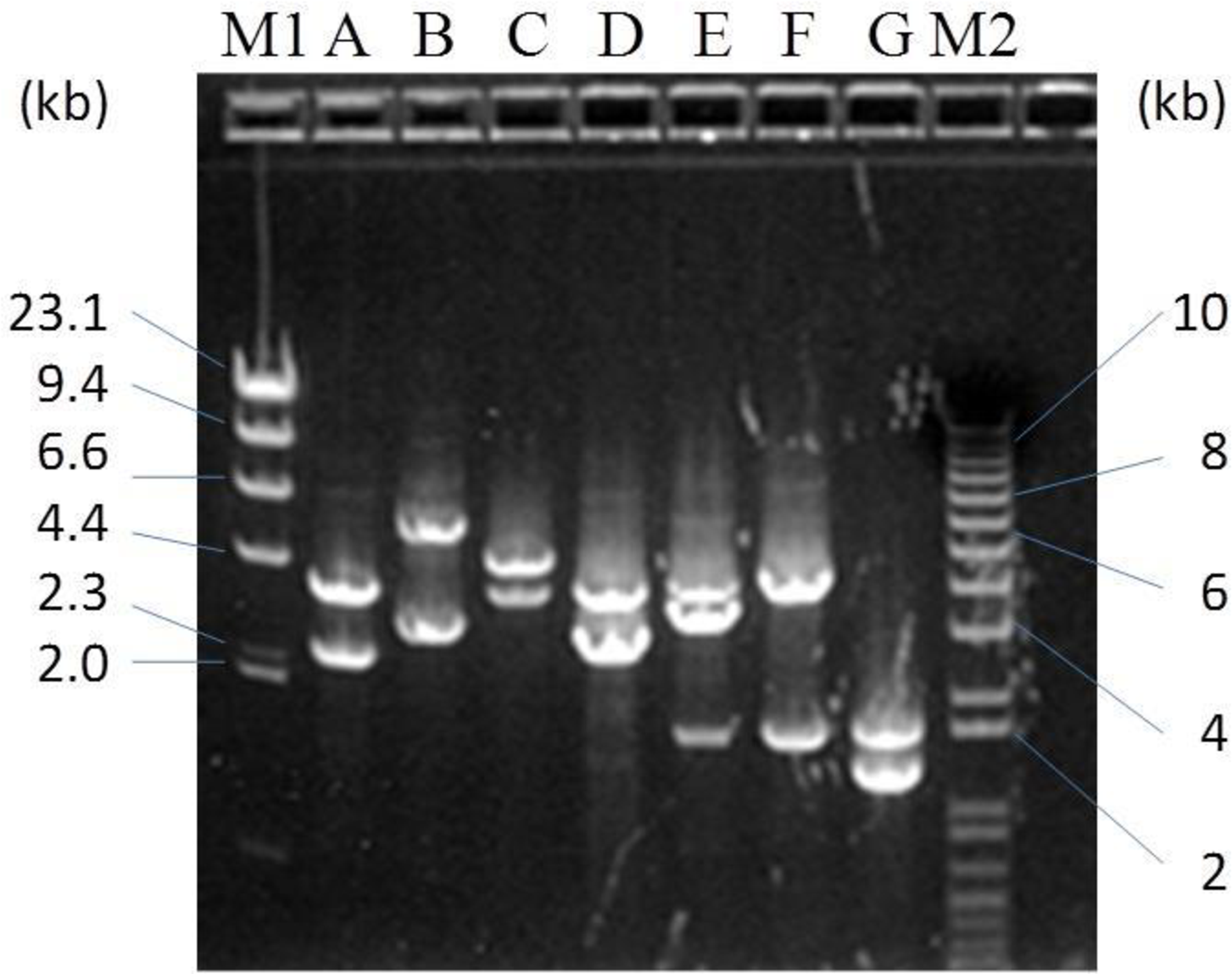

Supplement: S1 Fig — The LR-PCR products were electrophoresed on a 1% agarose gel. Each set of LR-PCR amplicons in a given lane had been amplified in the same well. All of the multiple LR-PCR reaction combinations were performed simultaneously using the same PCR machine. Lane M1: λDNA/HindIII marker; Lane A: combination A of LR-PCR product 2 (3,610 bp) and 12 (2,418bp); Lane B: combination B of LR-PCR products 4 (5,356 bp) and 17 (2,949 bp); Lane C: combination C of LR-PCR products 6 (3,555 bp) and 15 (4,516 bp); Lane D: combination D of LR-PCR products 1 (2,547 bp), 5 (2,579 bp), and 9 (3,559 bp); Lane E: combination E of LR-PCR products 3 (3,703 bp), 8 (3,079 bp), and 18 (1,516 bp); Lane F: combination F of LR-PCR products 7 (3,727 bp), 13 (4,172 bp), and 16 (1,534 bp); Lane G: combination G of LR-PCR products 10 (1,561 bp), 11 (1,240 bp), and 14 (1,206 bp); Lane M2: 1 kb DNA ladder (TAKARA Bio Inc, Kyoto, Japan). (TIF) [file pone.0166288.s001.tif]

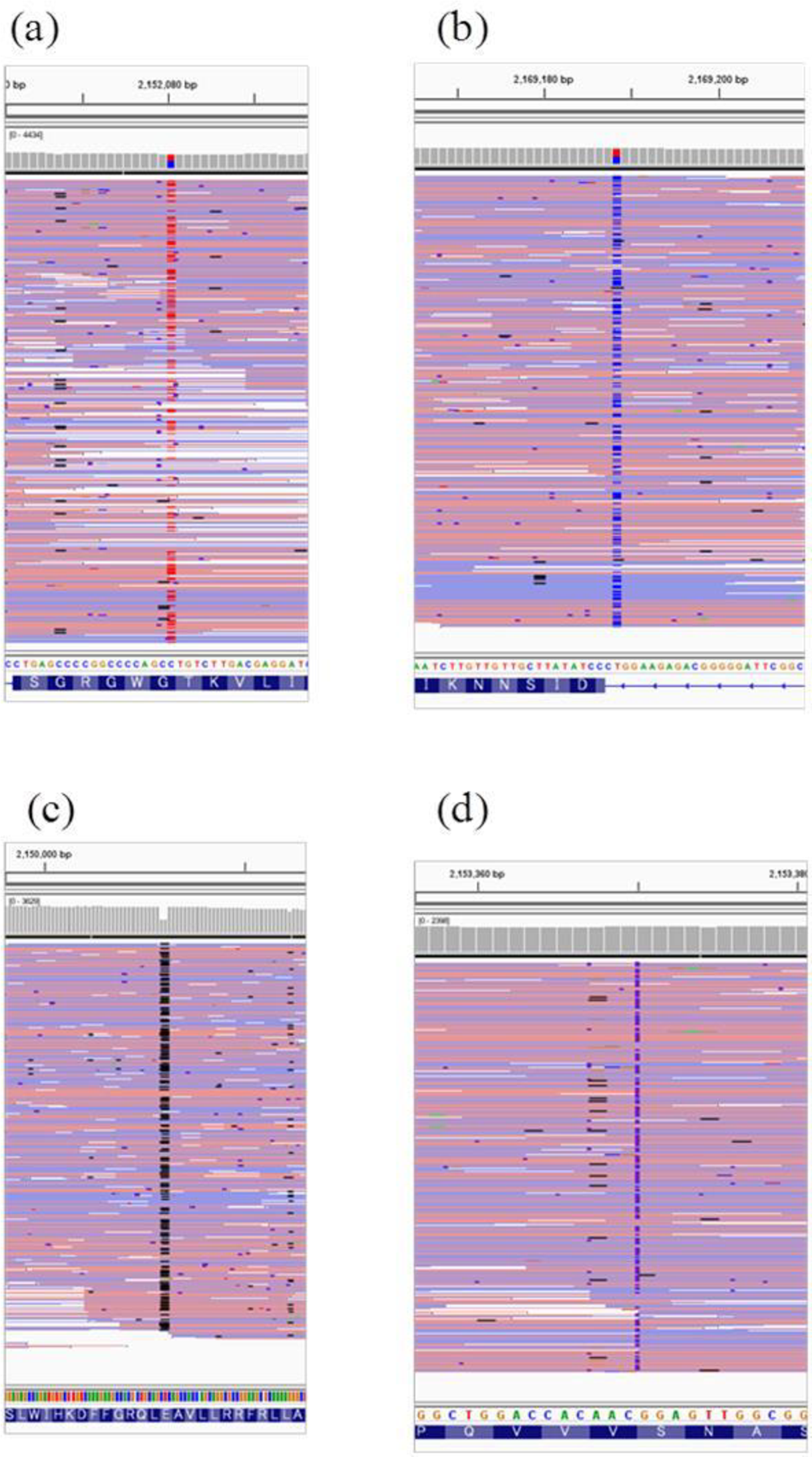

Supplement: S2 Fig — The upper gray bars show the depth of coverage and the lower red and blue lines show the mapped read fragments. (a) Missense mutation 9379G > A (G3127S) in PKD1, (b) splicing mutation IVS2-2A > G (D97_I120del24) in PKD2, (c) deletion 9755_9756delAG (E3252fs6X) in PKD1, and (d) insertion 8689insC (V2897fs39X) in PKD1. (TIF) [file pone.0166288.s002.tif]
